# Psoriasis and the Risk of Pneumonia: A Population-Based Study

**DOI:** 10.1371/journal.pone.0116077

**Published:** 2014-12-26

**Authors:** Li-Ting Kao, Cha-Ze Lee, Shih-Ping Liu, Ming-Chieh Tsai, Herng-Ching Lin

**Affiliations:** 1 Graduate Institute of Life Science, National Defense Medical Center, Taipei, Taiwan; 2 Sleep Research Center, Taipei Medical University Hospital, Taipei, Taiwan; 3 Department of Internal Medicine, National Taiwan University Hospital, Taipei, Taiwan; 4 Department of Urology, National Taiwan University Hospital and College of Medicine, Taipei, Taiwan; 5 Division of Gastroenterology, Department of Internal Medicine, Cathay General Hospital, Taipei, Taiwan; 6 School of Health Care Administration, Taipei Medical University, Taipei, Taiwan; University of Dundee, United Kingdom

## Abstract

**Background:**

Psoriasis is a prevalent autoimmune disorder. Various studies have reported on the relationship between psoriasis and chronic diseases but very few have explored the association between psoriasis and subsequent acute infection. This retrospective cohort study aimed to compare the risk of pneumonia between subjects with and those without psoriasis.

**Methods:**

The medical records of 14,022 patients with psoriasis and 14,022 without psoriasis were obtained from the Taiwan Longitudinal Health Insurance Database 2000. Each patient was followed-up for a three-year period. Cox proportional hazard regressions were performed to compare difference of subsequent pneumonia incidence between subjects with and those without psoriasis.

**Results:**

There were 206 (1.47%) subjects with psoriasis and 138 (0.98%) without psoriasis hospitalized for pneumonia. By Cox proportional hazard regressions analysis, the HR (hazard ratio) of pneumonia requiring hospitalization for patients with psoriasis was 1.50 (95% confidence interval [CI]: 1.21–1.86) compared to patients without psoriasis. The adjusted HR was 1.40 (95% CI: 1.12–1.73). The adjusted HR of pneumonia hospitalization for subjects with mild and severe psoriasis was 1.36 (95% CI: 1.09–1.70) and 1.68 (95% CI: 1.12–2.52), respectively, compared to those without psoriasis.

**Conclusions:**

Patients with psoriasis have significantly higher incidence of pneumonia compared to those without psoriasis.

## Introduction

Psoriasis is a chronic skin disorder known to be the most prevalent autoimmune disorder worldwide [1]. In the United States, psoriasis affects more than 2% of adults [2], while the prevalence in Europe varies from 0.6% to 6.5% in different regions [3]. Some literature report that psoriasis may be associated with immune system dysfunction and the production of pro-inflammatory cytokines [4]. These cytokines have been found to be etiologically involved in psoriasis and some common chronic diseases [4]–[7]. Various studies have demonstrated the relationship between psoriasis and chronic diseases like metabolic syndrome and chronic obstructive pulmonary disease (COPD) [8], [9]. However, very few have explored the association between psoriasis and acute infection.

Pneumonia is defined as an acute infection of the lung parenchyma [10]. It remains one of the leading causes of death and is commonly related with great morbidity, mortality, and utilization of healthcare resources [11], [12]. Recently, one study found a relationship between increased level of baseline pro-inflammatory cytokines such as TNF-α and IL-6 in the systemic circulation of elderly individuals, and an increased risk of subsequent community-acquired pneumonia requiring hospitalization [13]. It suggests that patients with psoriasis who are always in inflammatory states may have a higher incidence of acute infectious diseases than those without psoriasis. Nevertheless, the studies for this issue remain scant.

Two previous studies around the 1990s indicate that patients with psoriasis may have higher risk of viral infections, pneumonia, and sepsis [14], [15]. Another population-based study that included Dutch residents as the study population report that the likelihood of infectious diseases in patients with psoriasis have a two-fold higher risk of infection as the reference group [16]. However, no such studies have ever reported the association between psoriasis and pneumonia based on different psoriasis severities.

The aim of this population-based study was to investigate the relationship between psoriasis and subsequent risk of pneumonia. Moreover, this study also explored the risk of pneumonia on patients with varying psoriasis severity compared to the general population.

## Methods

### Database

Data for this population-based retrospective cohort study were taken from the Taiwan Longitudinal Health Insurance Database 2000 (LHID2000). Taiwan National Health Insurance (NHI) program, which was initiated in 1995, provides comprehensive and affordable medical care for all its citizens. The LHID2000 contains claims data of 1,000,000 individuals randomly selected from the 2000 Registry of Beneficiaries (n = 23.72 million) of the Taiwan NHI program. The LHID2000 allows researchers in Taiwan to follow-up all the medical services of these 1,000,000 enrollees since the beginning of Taiwan's NHI program. The LHID2000, which was open to the researchers in Taiwan, was available from the NHRI (http://nhird.nhri.org.tw/date_01.html). The high validity of data derived from the Taiwanese NHI program was demonstrated by many researchers and by the Taiwan National Health Research Institute [17], [18].

This study was exempt from full review by the Institutional Review Board of the National Defense Medical Center because the LHID2000 consists of de-identified secondary data released to the public for research purposes. This study did not use informed consent since all data were de-identified secondary data.

### Study Sample

**Figure 1:**
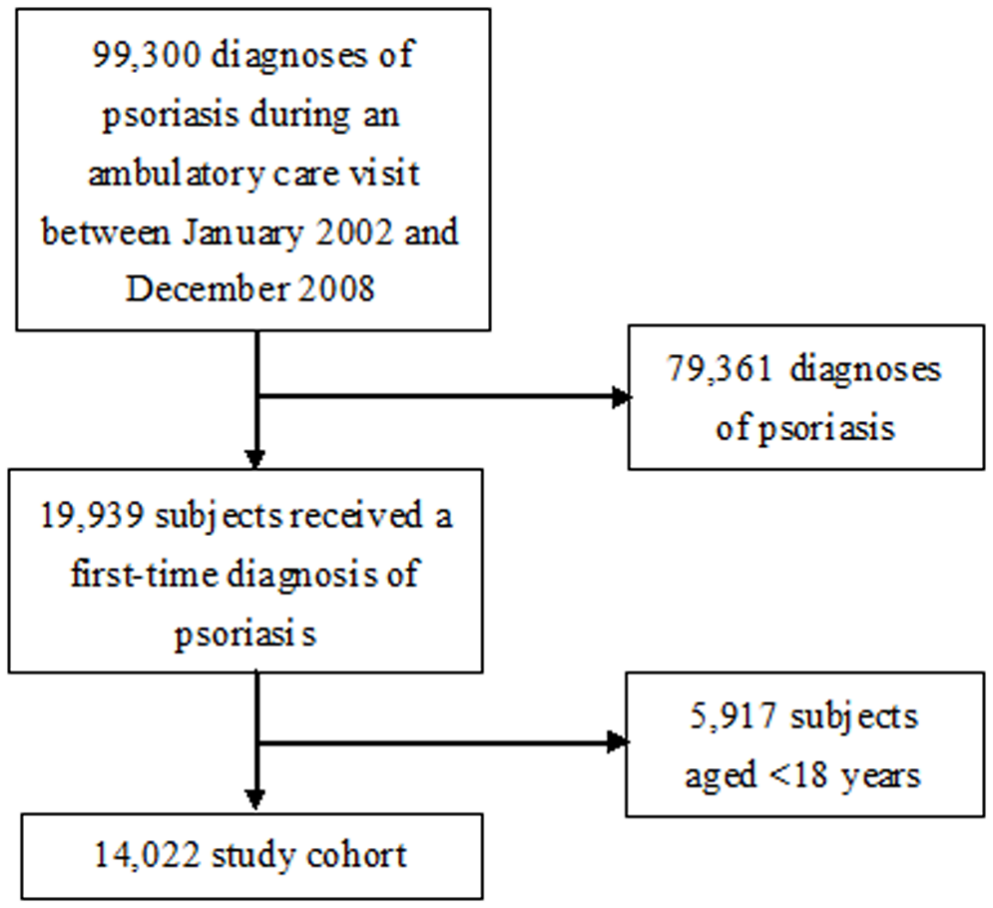
Flow diagram for study cohort.

This study features a study cohort and a comparison cohort. The study cohort included 19,939 subjects who received a first-time diagnosis of psoriasis (ICD-9-CM code 696 or 696.1) during an ambulatory care visit between January 2002 and December 2008. The date of the first diagnosis of psoriasis was set as the index date. None of the subjects had ever received a diagnosis of psoriasis prior to the index date. Those who were aged <18 years (*n* = 5,917) were further excluded to limit the subjects to the adult population. As total of 14,022 subjects with psoriasis were finally included as the study cohort. The selection procedures were shown in Fig. 1.

The matched comparison cohort (*n* = 14,022) (one comparison subject per patient with psoriasis) were also retrieved from the remaining beneficiaries of the LHID2000. This comparison cohort was selected by matching patients with psoriasis in terms of gender, age group (18–24, 25–34, 35–44, 45–54, 55–64, 65–74, 75–84, and ≥85 years), and year of the index date. For the comparison cohort, the year of the index date was simply a matched year when the comparison subjects had a medical utilization. In addition, for comparison subjects, the date of their first use of ambulatory care during that matched year was set as the index date. None of the selected comparison subjects had received a diagnosis of psoriasis since the initiation of the NHI program in 1995.

The patients with psoriasis were further classified into two cohorts based on psoriasis severity. Those who received the systemic therapy, including oral retinoids, methotrexate, cyclosporine, ultraviolet B (UVB) phototherapy or photo-chemotherapy, and biologicals were defined as patients with severe psoriasis (n = 1,511 or 10.8% if all patients with psoriasis). Those who did not received any systemic therapy were regarded as patients with mild psoriasis (*n* = 12,511; 89.2%).

Each patient was individually tracked for three years from their index date to determine all of those who were hospitalized with a principal diagnosis of pneumonia (ICD-9-CM 480–483, 485–486, and 487.0). This study included all types of pneumonia including pneumococcal pneumonia, viral pneumonia, influenza pneumonia and bacterial pneumonia.

### Statistical Analysis

All analyses were conducted using the SAS system (SAS System for Windows, version 8.2, SAS Institute, Cary, NC). Chi-square test and Pearson χ^2^ test were performed to compare differences between the study and comparison cohort in terms of the patients' monthly income, geographic location (i.e., northern, central, eastern, and southern Taiwan), urbanization level (five levels, with 1 being the most urbanized and 5 being the least), and selected medical co-morbidities including hypertension, diabetes mellitus, chronic obstructive pulmonary disease (COPD), renal failure, liver diseases and neurological diseases (migraines, headaches and Parkinson's disease), rheumatologic diseases (rheumatoid arthritis, systemic lupus erythematosus and ankylosing spondylitis), alcohol abuse, and tobacco use disorder. These selected co-morbidities were only included if they were diagnosed before the index date. The Kaplan-Meier method and log-rank test were performed to estimate the three-year pneumonia-free survival rate.

We also calculated the propensity score for each patient included in this study. A propensity score was used to balance demographic characteristics (monthly income, geographic location and urbanization level) and co-morbidities (hypertension, diabetes mellitus, COPD, renal failure, liver diseases, neurological diseases, rheumatologic diseases, alcohol abuse and tobacco use disorder), which were distributed unequally between patients with psoriasis and those without psoriasis at baseline. Subsequently, Stratified Cox proportional hazard regression analysis was performed to calculate the hazard ratio (HR) of pneumonia during the three-year follow-up period between the two cohorts after adjusting for propensity score. We presented hazard ratios (HR) along with 95% confidence interval (CI). Statistical significance was set at a two-sided *p*<0.05.

## Results

The study cohort comprised 14,022 patients with psoriasis and 14,022 matched comparison cohort. The 28,044 patients in the study sample had a mean age of 41.1±17.6 years (±standard deviation). The demographic characteristics and selected medical co-morbidities of patients with and those without psoriasis were presented in Table 1. After matching for gender, age, and year of the index date, there were significant differences in monthly income (*p* = 0.006) and geographic region (*p*<0.001) between the study and comparison cohort. The study cohort had higher prevalences of co-morbidities of hypertension (25.3% vs. 22.1%; *p*<0.001), diabetes (14.9% vs. 12.1%; *p*<0.001), COPD (1.7% vs. 1.1%; *p*<0.001), renal failure (3.1% vs. 2.1%; *p*<0.001), liver diseases (21.8% vs. 17.5%; *p*<0.001), neurological diseases (42.8% vs. 40.2%; *p*<0.001), rheumatologic diseases (6.4% vs. 4.2%; *p*<0.001) and alcohol abuse (1.0% vs. 0.7%; *p* = 0.009) than the comparison cohort.

**Table 1:** Demographic characteristics and co-morbidities of subjects with and those without psoriasis (n = 28,044).

Variable	Patients with psoriasis (n = 14,022)		Comparison subjects (n = 14,022)		*p* value
	Total no.	%	Total no.	%	
Monthly Income					0.006
NT$ 1–15,841	5,180	36.9	5,189	37.0	
NT$ 15,841–25,000	4,528	32.3	4,736	33.8	
≥NT$ 25,001	4,314	30.8	4,097	29.2	
Geographic region					<0.001
Northern	6,834	48.7	6,992	49.9	
Central	3,021	21.5	3,237	23.1	
Southern	3,859	27.5	3,523	25.1	
Eastern	308	2.2	270	1.9	
Urbanization level					0.647
1 (most urbanized)	4,516	32.2	4,482	32.0	
2	3,560	25.4	3,652	26.0	
3	2,291	16.3	2,313	16.5	
4	1,810	12.9	1,789	12.8	
5 (least urbanized)	1,845	13.2	1,786	12.7	
Monthly Income					0.006
NT$ 1–15,841	5,180	36.9	5,189	37.0	
NT$ 15,841–25,000	4,528	32.3	4,736	33.8	
≥NT$ 25,001	4,314	30.8	4,097	29.2	
Hypertension	3,546	25.3	3,098	22.1	<0.001
Diabetes	2,093	14.9	1,693	12.1	<0.001
Chronic obstructive pulmonary disease (COPD)	235	1.7	158	1.1	<0.001
Renal failure	432	3.1	295	2.1	<0.001
Liver diseases	3,053	21.8	2,453	17.5	<0.001
Neurological diseases	6,001	42.8	5,641	40.2	<0.001
Rheumatologic diseases	896	6.4	589	4.2	<0.001
Alcohol abuse	139	1.0	99	0.7	0.009
Tobacco use disorder	292	2.1	281	2.0	0.642

**Figure 2:**
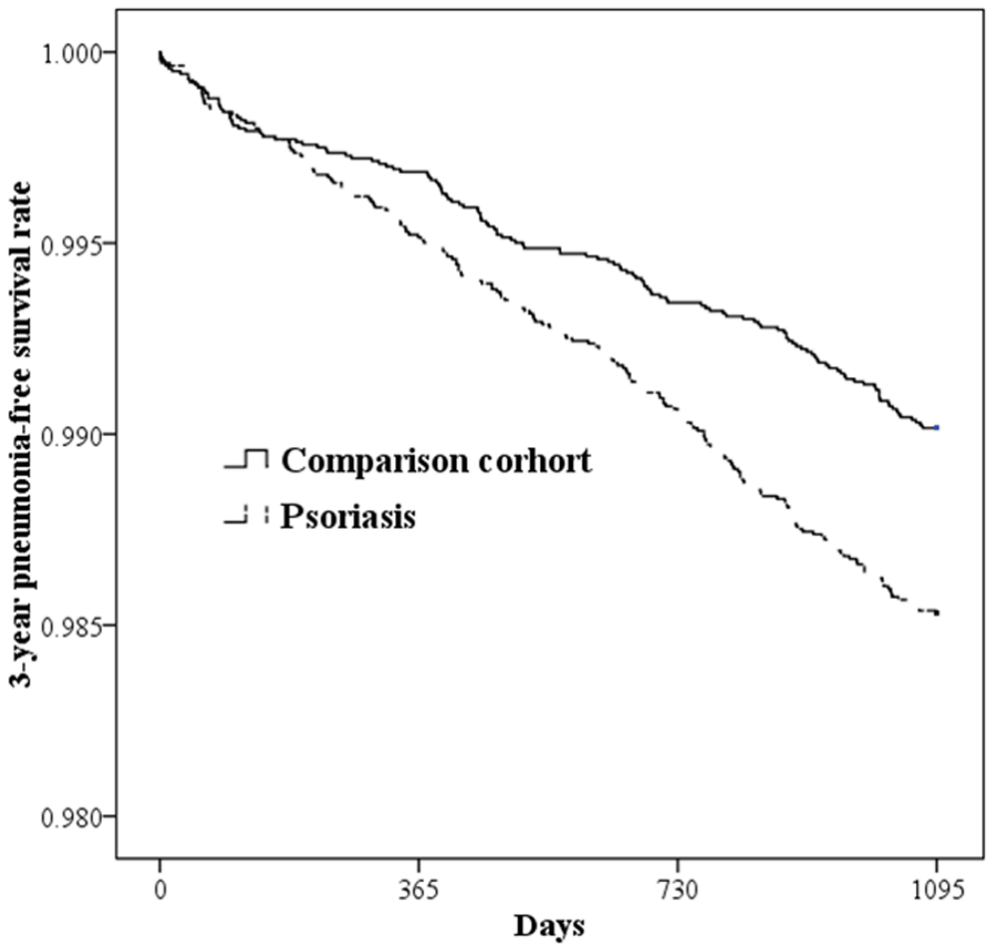
Three-year pneumonia-free survival rates for patients with psoriasis and comparison cohort.

The prevalence and HR of pneumonia requiring hospitalization within three years after the index date between the study and comparison cohorts (Table 2) revealed that 344 (1.23%) of total sampled patients were subsequently hospitalized for pneumonia. Subsequent hospitalization of pneumonia was found in 206 (1.47%) patients with psoriasis and in 138 (0.98%) without psoriasis. Of the sampled patients requiring hospitalization, the length of stay was 9.4 and 9.3 days for the study and comparison cohorts, respectively (*p* = 0.854). The log-rank test shows a significant difference in pneumonia-free survival rates between patients with and without psoriasis during the three-year follow-up period (*p*<0.001). Fig. 2 displays the survival times for both two cohorts based on Kaplan-Meier survival analysis.

**Table 2:** Prevalence, hazard ratios (HRs), and 95% confidence intervals (CIs) for pneumonia requiring hospitalization after the index date within three years among the study population.

Following incidence of pneumonia requiring hospitalization	Total (n = 28,044)	Subjects with psoriasis (*n* = 14,022)	Controls (*n* = 14,022)
	n, %	n, %	n, %
Yes	344 (1.23)	206 (1.47)	138 (0.98)
No	27,700 (98.77)	13,816 (98.53)	13,884 (99.02)
Crude HR (95% CI)	—	1.50*** (1.21∼1.86)	1.00
Adjusted HR (95% CI) a	—	1.40** (1.12∼1.73)	1.00

Notes: The adjusted HR was calculated by Cox proportional hazard regression stratified by sex and age group.

a Adjustment for propensity score.

***p<0.001; **p<0.01.

The Cox proportional hazard regression analysis indicated that the crude HR of pneumonia requiring hospitalization for patients with psoriasis was 1.50 (95% confidence interval [CI]: 1.21–1.86) than those without psoriasis. After adjusting for propensity score, those with psoriasis were more likely to be hospitalized for pneumonia (HR: 1.40; 95% CI: 1.12–1.73). The prevalence of pneumonia requiring hospitalization within three years after the index date between patients with mild and those with severe psoriasis, and those without psoriasis (Table 3) revealed that patients with mild psoriasis were more likely to be hospitalized for pneumonia than those without psoriasis (HR: 1.43; 95% CI: 1.15–1.79), even after adjusting for propensity score (HR: 1.36; 95% CI: 1.09–1.70). Patients with severe psoriasis had higher risk of being hospitalized for pneumonia within three years after the index date compared to subjects without psoriasis (HR: 2.03; 95% CI: 1.37–3.01). Furthermore, the HR for subjects with severe psoriasis was 1.68 (95% CI: 1.12–2.52) in comparison to subjects without psoriasis after adjusting for propensity score.

**Table 3:** Prevalence, hazard ratios (HRs), and 95% confidence intervals (CIs) for pneumonia requiring hospitalization after the index date within three years among the study population, by psoriasis severity.

Following incidence of pneumonia requiring hospitalization	Subjects with severe psoriasis (n = 1,511)	Subjects with mild psoriasis (n = 12,511)	Comparison subjects (n = 14,022)
	n, %	n, %	n, %
Yes	30 (1.99)	176 (1.41)	138 (0.98)
No	1,481 (98.01)	12,335 (98.59)	13,884 (99.02)
Crude HR (95% CI)	2.03*** (1.37∼3.01)	1.43** (1.15∼1.79)	1.00
Adjusted HR (95% CI) a	1.68*** (1.12∼2.52)	1.36*** (1.09∼1.70)	1.00

Notes: The adjusted HRs were calculated by Cox proportional hazard regression stratified by sex and age group.

a Adjustments for propensity score.

***p<0.001; **p<0.01.

## Discussion

This population-based study investigated the association between psoriasis and pneumonia. Patients with psoriasis were 1.50-times (adjusted HR: 1.40) more likely to be hospitalized for pneumonia than patients without psoriasis. To date, few studies mention the association of psoriasis with infection. The relationship between psoriasis and pneumonia according to the severity of psoriasis remains unclear. One study in 1996 explored five cases with psoriasis complicated by Staphylococcal septicemia [15]. Another study in Switzerland published in 1986 found that patients with psoriasis showed significantly higher rates of viral infections and pneumonia than healthy controls [14]. A recent study in the Netherlands of 25,742 subjects with psoriasis and 128,710 subjects without psoriasis also demonstrated a relationship between psoriasis and infectious disease.^16^ Subjects with psoriasis were more likely to acquire infectious diseases than those without psoriasis (hazard ratio [HR] 2.08, 95% CI: 1.96–2.22). In addition, lower respiratory tract infections happened more frequently in the study cohort than in the comparison (crude HR 2.37, 95% CI: 2.09–2.68; adjusted HR [aHR] 1.22; 95% CI: 1.07–1.39).

The results in this study may be explained by the aggravation of the immune function and the production of the pro-inflammatory cytokines in patients with psoriasis. Psoriasis is well categorized as an immune-mediated inflammatory disease [4], [19]. Numerous studies have shown that serum levels of pro-inflammatory cytokines like tumor necrosis factor-α (TNF-α), interleukins, and interferon-γ were significantly higher in patients with psoriasis than in controls [20]–[22]. Moreover, one study in the United States report an association between increased baseline levels of TNF-α and IL-6 in the systemic circulation and high risk of subsequent hospitalization due to community-acquired pneumonia (CAP) [13]. It also demonstrates that the highest tertile of TNF and IL-6 levels are related to CAP susceptibility, with adjusted ORs of 1.6 (95% CI: 1.0–2.7) and 1.7 (95% CI: 1.1–2.8). Therefore, elevated serum levels of pro-inflammatory cytokines in patients with psoriasis may be the potential reason for their higher incidence of pneumonia in this study.

This study further explored the relationship between psoriasis and pneumonia based on psoriasis severity. Those with mild psoriasis were 1.43-times (adjusted HR: 1.36) more likely to be hospitalized of pneumonia than those without psoriasis. Patients with severe psoriasis were 2.03-times (adjusted HR: 1.68) more likely to be hospitalized for pneumonia than the controls. Patients with severe psoriasis seemed to have a higher risk of pneumonia than those with mild psoriasis. Only one previous study in the Netherlands report a similar relationship between infection and psoriasis [16].

The study cohort using systemic therapy were 2.54-times (95% CI: 2.21–2.90) more likely to acquire infectious diseases requiring hospitalization compared to the comparison cohort [16]. Compared to the comparison cohort, the study cohort using topical medicine was 2.01-times (95% CI: 1.88–2.15) more likely to acquire infectious diseases requiring hospitalization. Several reasons may explain the high likelihood of pneumonia requiring hospitalization among patients with severe psoriasis. First, high levels of cytokines are correlated with the clinical severity of psoriasis [21]. Serum cytokine levels have been associated with the incidence of pneumonia [13]. Second, patients with severe psoriasis may receive systemic therapy, including methotrexate and cyclosporine. Some systemic medications may have immuno-suppressive effects and increase the risk of infections [23]–[25]. However, we cannot confirm whether it was the psoriasis or the treatment that may contribute to the increasing risk of pneumonia in the severe psoriasis group. Lastly, patients with severe psoriasis may have increased prevalence of various co-morbidities [26]–[28], which may affect susceptibility to infections.

A specific strength of this study is the use of a nationwide population-based dataset with generous health benefit coverage in Taiwan. This feature increases the statistical power and affords sufficient sample size to identify the relationship between psoriasis and pneumonia after adjusting for several confounders. Moreover, this characteristic can decrease the effects of selection bias. Nonetheless, there are also several limitations that need to be considered. First, the NHI Research Database used in this study does not offer information regarding lifestyle factors or health habits, such as smoking, weight gain, and contact with pets, which are all risk factors for pneumonia that may affect the association between psoriasis and pneumonia [29], [30]. Second, LHID2000 does not provide the information on data for laboratory examination, microbial etiology, CURB-65 pneumonia severity score, Psoriasis Area Severity Index (PASI) and pneumonia mortality. However, although our database did not provide the PASI, the severity of psoriasis in the present study has been categorized according to the prescriptions of systemic therapy. This definition has been frequently used in other prior studies [31]–[33]. Finally, it is also possible that the database does not contain all of the patients with psoriasis. Some with mild psoriasis may not seek health services that are covered by the NHI program because patients consider the related treatments as inessential.

In summary, this population-based study demonstrates that subjects with psoriasis are associated with increased risk of subsequent pneumonia requiring hospitalization. Patients with severe and mild psoriasis have higher risk of pneumonia requiring hospitalization than those without psoriasis. The risk of pneumonia incidence in patients with severe psoriasis is higher than in those with mild psoriasis. Nevertheless, further studies are warranted to determine the real mechanisms involved in the relationship between psoriasis and pneumonia.
